# Management of an Intralingual Ancient Schwannoma

**DOI:** 10.7759/cureus.115770

**Published:** 2026-09-04

**Authors:** Harris Syed, Bailey Balouch, Upasana Joneja, Yekaterina Shapiro

**Affiliations:** 1 Otolaryngology - Head and Neck Surgery, Cooper Medical School of Rowan University, Camden, USA; 2 Otolaryngology - Head and Neck Surgery, Cooper University Hospital, Camden, USA; 3 Pathology, Cooper University Hospital, Camden, USA

**Keywords:** ancient schwannoma, head and neck tumor, intralingual schwannoma, lingual mass, tongue neoplasm

## Abstract

Schwannomas are benign tumors arising from Schwann cells. Lingual schwannomas are exceedingly rare and usually present as painless, slow-growing masses, usually of the oral tongue or tongue base, with symptoms such as dysphagia, discomfort, and, in some cases, airway compromise. The standard of care is surgical excision via a transoral approach. In this case, we discuss an intralingual ancient schwannoma presenting with dysphagia, discomfort, and throat fullness that was subsequently excised with excellent functional outcomes.

## Introduction

Schwannomas are benign, slow-growing peripheral nerve sheath tumors that often arise in the head and neck. Most commonly, these tumors originate from the vagus nerve and sympathetic trunk [1]. Ancient schwannomas are a degenerative variant characterized by hypocellularity, calcification, pleomorphism, microcystic change, dilated vessels, and hemorrhage [1,2]. Intralingual ancient schwannomas of the head and neck are uncommon, and symptoms usually result from mass effect with the neoplasm compressing adjacent structures [1]. Symptomatic or enlarging schwannomas are typically surgically removed, whereas stable asymptomatic lesions may be observed [3-6]. Malignant transformation of surgically excised symptomatic cases is exceptionally rare [1,2].

Given the nonspecific symptoms of these rare tumors, diagnosis may be challenging due to a wide differential that must be considered. This case highlights the clinical, radiological, and histopathological characteristics of an ancient intralingual schwannoma while demonstrating a successful operative treatment strategy. Moreover, the ambiguity of this case allows for a thorough discussion of the differential and potential treatment strategies involved when considering an intralingual mass. Awareness of masses such as those in this case may guide clinicians when working up these nonspecific symptoms, allowing for more efficient treatment.

## Case presentation

History

A 69-year-old Caucasian female with a past medical history of hypertension, thyroid disease, osteoporosis, and polymyalgia rheumatica presented for evaluation of a 2.5 x 2.0 x 1.2-cm midline lingual mass incidentally found on MRI obtained for headaches. She reported feeling a small lump on the right side of her throat with occasional dysphagia for about 10 years leading up to these imaging findings. She denied odynophagia, voice changes, or constitutional symptoms.

Physical examination

On physical examination, the mass was not palpable, and head, eyes, ears, nose, and throat (HEENT) exam was unremarkable. Additionally, the mass was not visualized on flexible fiberoptic nasopharyngolaryngoscopy, making biopsy in the clinic infeasible.

Diagnostic Evaluation

As seen in Figure 1, the mass was hypointense on T1-weighted imaging, hyperintense and heterogeneous on T2-weighted imaging, and interposed between the paired genioglossus muscles. At this point, an epidermoid cyst was the leading differential, with ear, nose, and throat (ENT) department follow-up as the next step in management. Percutaneous biopsy was also deemed infeasible by interventional radiology.

**Figure 1:**
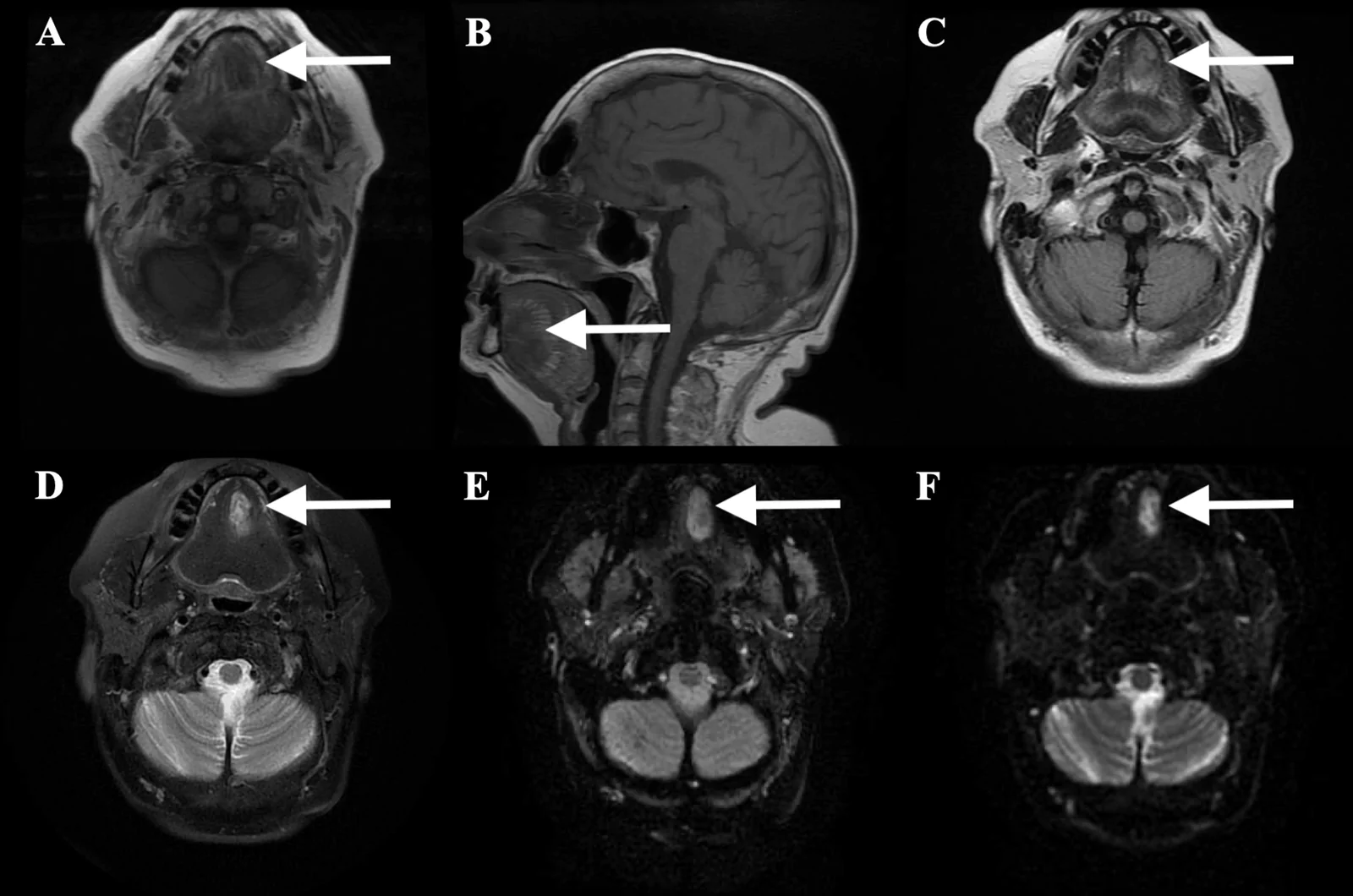
MRI demonstrating the intralingual mass The lesion is center in the midline of the tongue between the paired genioglossus muscles (arrows). The mass demonstrates low signal intensity on T1-weighted imaging and heterogeneous high signal intensity on T2-weighted imaging.

Management

The patient and surgeon agreed to proceed with excision for diagnostic and therapeutic purposes. The patient was taken to the operating room, and the ventral surface of the lingual mucosa was incised in the midline. The underlying ventral tongue musculature was dissected in the midline until the mass was encountered (Figure 2). The mass was dissected circumferentially and removed in its entirety. Permanent histopathology of the lesion was sent rather than intra-operative frozen section. It is unclear as to the reasoning behind not obtaining the frozen section; however, the leading diagnosis up to this point was benign epidermoid cyst. Additionally, the pathology report did not explicitly mention negative surgical margins. The wound edges were reapproximated in layers.

**Figure 2:**
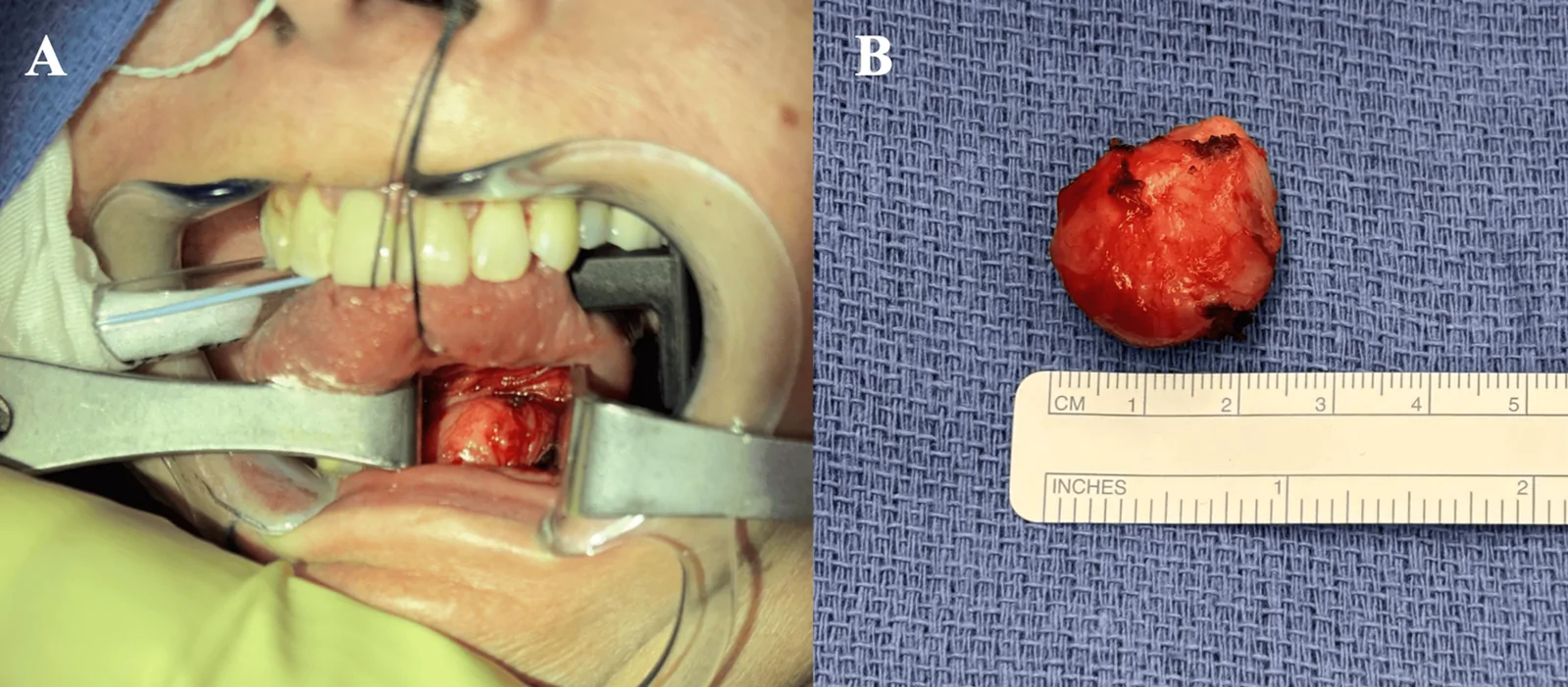
Intraoperative resection and gross appearance of the intralingual mass (A) Intraoperative view with ventral exposure of the tongue and midline mass following dissection of the lingual musculature; (B) Approximately 2.5 cm tan soft tissue mass resected

As seen in Figure 3 and Figure 4, pathology confirmed a benign peripheral nerve sheath tumor with extensive hyalinization and ancient change supporting the diagnosis of ancient intralingual schwannoma (Figure 3). Moreover, immunohistochemical staining resulted in cells positive for S100 and negative for CD34 and smooth muscle actin (Figure 5). Congo red stain was also negative for amyloid. Although the rationale behind obtaining Congo red staining for amyloid was not included in the surgical pathology report, it is reasonable to infer that the prominent hyalinization seen on microscopy may have prompted further investigation to rule out an amyloid-depositing process.

**Figure 3:**
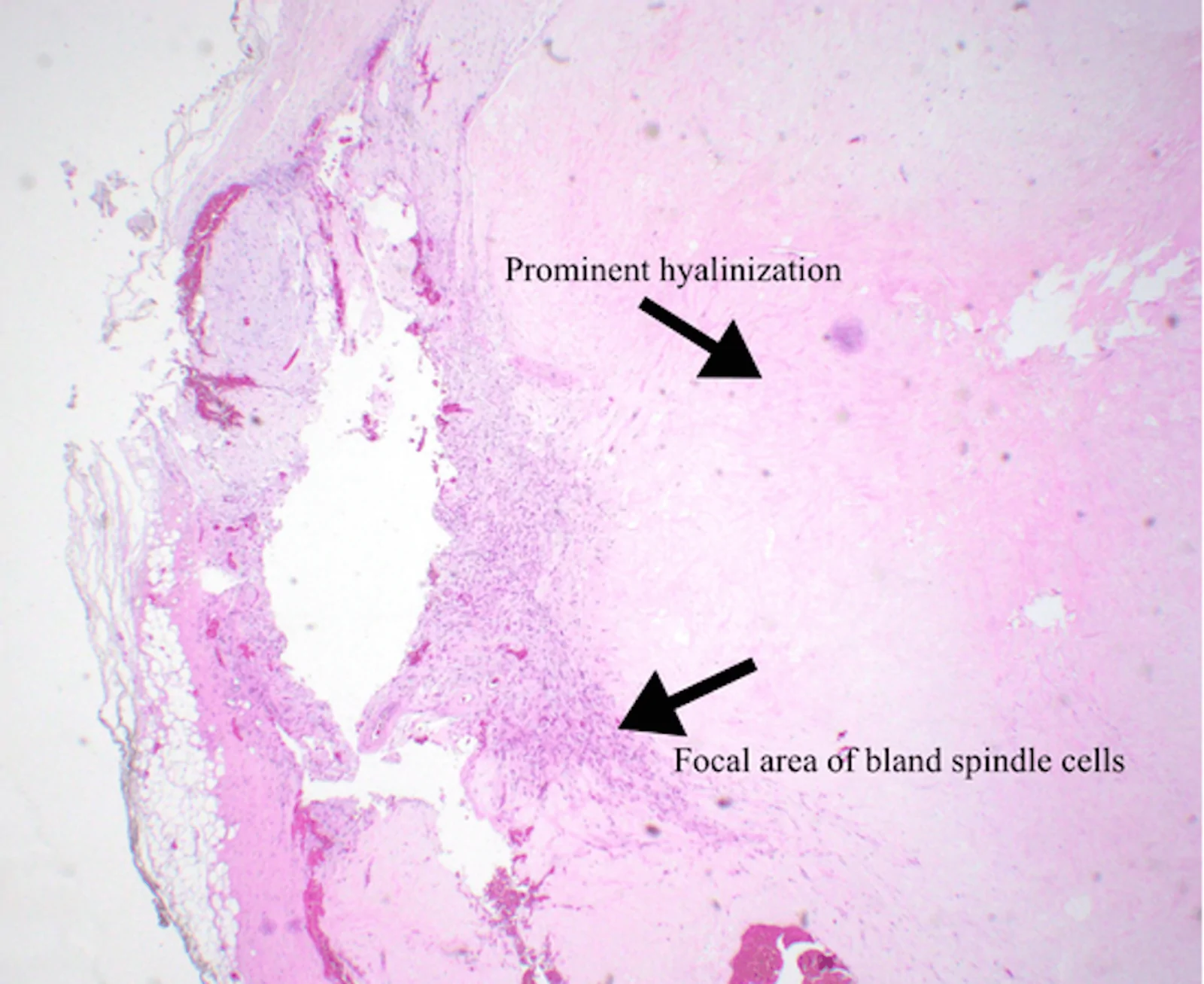
H&E 20x magnification The image shows a well-circumscribed mass that is predominantly hyalinized with a focal area of bland spindle cells.

**Figure 4:**
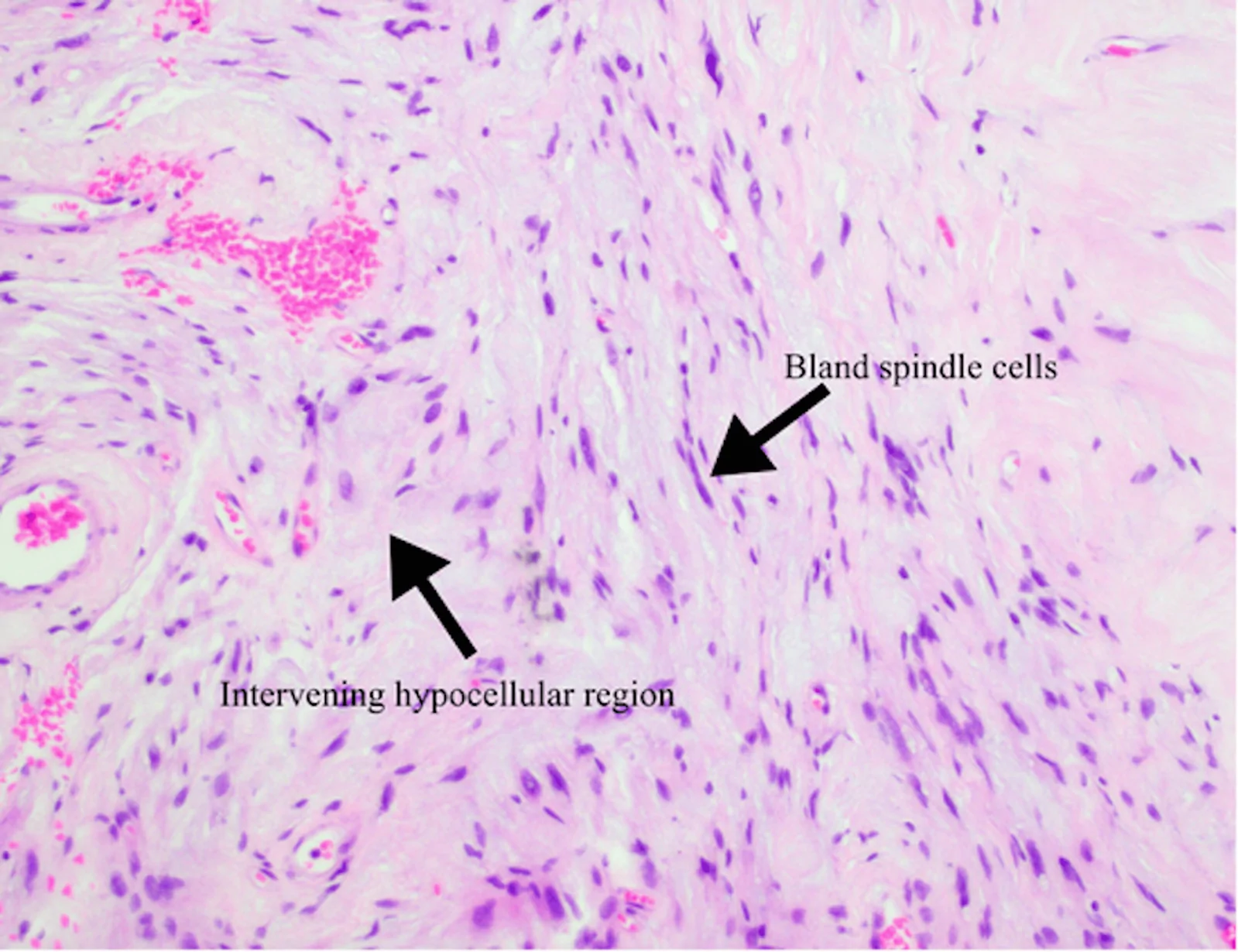
H&E 200x magnification The cellular areas were composed of bland spindle cells with intervening hypocellular regions. Mitotic activity was low.

**Figure 5:**
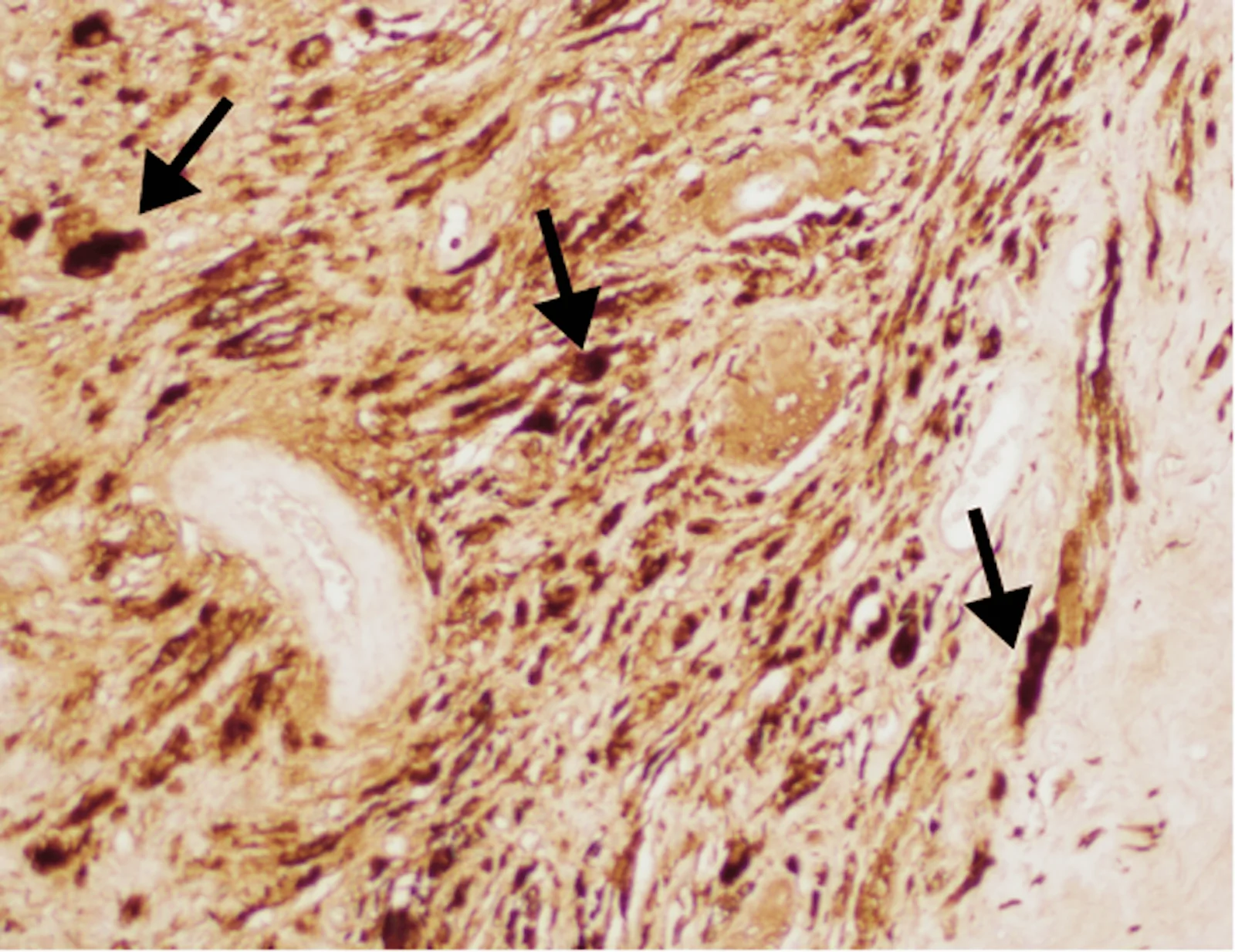
S100 immunohistochemical stain, 200x magnification The bland spindle cells were diffusely positive for S100, and CD34 was negative, supporting the diagnosis of schwannoma. The arrows highlight S100-positive and CD34-negative spindle cells.

Outcomes and follow-up

The patient was seen in follow-up in two weeks. Her wound healed uneventfully with full preservation of tongue sensation and mobility. Her symptoms have resolved completely. The patient was not seen again after the two-week follow-up.

## Discussion

According to the current literature on schwannomas, 1% of all head and neck schwannomas are intralingual, and the ancient schwannoma subtype is exceedingly uncommon [7-9]. A 20-year retrospective series of 40 oral and maxillofacial schwannomas demonstrated only eight cases (20%) of ancient subtypes with tongue involvement [10]. Schwannomas are typically solitary lesions but can occur as a part of syndromes such as neurofibromatosis and schwannomatosis, yet a specific syndromic association with intralingual schwannomas has not been clearly established [11]. Although these neoplasms are rare, surgical excision remains the standard of care for symptomatic or growing schwannomas [3-5].

The differential diagnosis for a lingual mass includes mucocele, ranula, lingual thyroid, dermoid cysts, and epidermoid cysts [12-14]. Mucoceles and ranulas are benign mucus cavity lesions that may present as painless oral swellings and are typically treated with excision [12]. Lingual thyroid is characterized by the abnormal presence of thyroid tissue at the base of the tongue that may cause dysphagia or airway symptoms; management depends on symptom severity and thyroid function, with observation of thyroid hormone suppression in mild cases and surgery in more severe cases [13]. Dermoid and epidermoid cysts are slow-growing developmental lesions that are generally asymptomatic but may require surgical excision [14]. Squamous cell carcinoma of the tongue must also be considered in persistent or enlarging tongue masses because these malignant lesions require prompt biopsy and oncologic management [15].

The surgical approach for resection of intra-oral masses depends on lesion size, location, and exposure. For tongue base lesions, transoral robotic surgery (TORS) offers precise dissection with excellent hemostasis [16]. More extensive or laterally located lesions may require lateral pharyngotomy, while very large or infiltrative tumors may require mandibulotomy-based approaches. These approaches provide greater exposure but carry higher morbidity [17].

Mass location warrants intraoperative caution. Lingual schwannomas frequently involve the hypoglossal nerve, and operative injury may result in ipsilateral tongue weakness, atrophy, and deviation. Clinical symptoms such as dysarthria, dysphagia, and impaired tongue mobility may persist and negatively affect a patient’s functional status [1,18]. Despite the risk for iatrogenic nerve injury, complications are rare, and functional outcomes are consistently exceptional.

Resection also carries risks of hemorrhage, hematoma, and infection. Since the tongue is highly vascularized, meticulous hemostasis is essential; however, serious complications are uncommon [19]. Additionally, many studies have shown that recurrence of these lesions following resection is exceedingly rare. Specifically, Cohen and Wang found no reported recurrences following surgical excision in a review of 126 reported tongue schwannomas [11]. Moreover, Cohen and Wang note that malignant transformation of these intralingual schwannomas is highly uncommon as well, although an exact percentage is not specified. The patient was doing well at her two-week postoperative appointment with resolution of her symptoms. The surgeon felt that observation was a reasonable surveillance method in this case. It has been about 12 months since the operation, and the patient has not followed up after her two-week postoperative appointment.

The primary clinical value of this case centers on the diagnostic uncertainty regarding an intralingual mass with nonspecific symptoms and imaging findings. In this case, the lesion was initially thought to be an epidermoid cyst. Physical exam and flexible fiberoptic nasopharyngolaryngoscopy did not provide a tissue sample for lesion characterization. Definitive diagnosis and treatment required total excision of the mass, resulting in complete resolution of the patient's symptoms. This case highlights the importance of keeping a wide differential when working up these lesions, particularly when physical exam and radiologic imaging are nonspecific. Moreover, this case demonstrates the utility of the current midline translingual approach in treatment.

## Conclusions

Intralingual ancient schwannoma is a rare benign peripheral nerve sheath tumor that may present with dysphagia, throat fullness, or other symptoms related to mass effect. Surgical excision remains the preferred treatment for symptomatic lesions and can provide excellent functional outcomes. Midline translingual access may be an effective approach for centrally located intralingual tumors with favorable exposure and preservation of function.
